# (mis)-Targeting of SWI/SNF complex(es) in cancer

**DOI:** 10.1007/s10555-023-10102-5

**Published:** 2023-04-24

**Authors:** Divya Reddy, Saikat Bhattacharya, Jerry L. Workman

**Affiliations:** Stowers Institute for Medical Research, Kansas City, MO 64110 USA

**Keywords:** BAF complex, Chromatin remodeling, Epigenetics, Mutations, Therapy, Inhibitors

## Abstract

The ATP-dependent chromatin remodeling complex SWI/SNF (also called BAF) is critical for the regulation of gene expression. During the evolution from yeast to mammals, the BAF complex has evolved an enormous complexity that contains a high number of subunits encoded by various genes. Emerging studies highlight the frequent involvement of altered mammalian SWI/SNF chromatin-remodeling complexes in human cancers. Here, we discuss the recent advances in determining the structure of SWI/SNF complexes, highlight the mechanisms by which mutations affecting these complexes promote cancer, and describe the promising emerging opportunities for targeted therapies.

## Introduction

Cancer is a genetic disease arising due to the various mutations in the DNA sequence that encodes key proteins essential for the functioning of the cell. The DNA sequence, therefore, receives a lot of attention, and rightly so. However, the DNA inside the cell is not naked. It is packaged with proteins in the form of chromatin. Hence, the cells need to have a mechanism by which the underlying DNA sequence can be made accessible to machinery such as those of transcription and repair. This is brought about largely by protein complexes known as chromatin remodelers—“remodelers” because they displace or evict the histone protein octamers around which the DNA is wrapped for packaging [1]. There are a variety of chromatin-remodeling protein complexes such as the SWI/SNF, CHD1, and RISC among others [1]. In this review, we will restrict our focus to the SWI/SNF complex because of its specific involvement in cancer.

SWI/SNF is an ATP-dependent, multi-subunit, chromatin remodeling complex. It uses the energy from ATP hydrolysis to perform its remodeling function. SWI/SNF subunits were first identified in yeast as genes essential for enabling mating-type *swi*tching and sucrose metabolism (*S*ucrose *n*on-*f*ermenting) [2, 3]. Many of the proteins encoded by these genes were later found to be part of a multi-subunit protein assembly that was named the SWI/SNF complex [4–7]. This 11-subunit complex (in yeast) has a DNA-stimulated ATP-dependent activity and it acts on the chromatin template [8]. It disrupts histone–DNA contacts, resulting in the sliding of the histone octamer on DNA or the eviction of histones making the DNA accessible for binding of various transcription factors [9, 10]. Perhaps the most essential component of this complex is the ATPase while the other subunits aid in either activating or recruitment of the complex on chromatin. Consistent with its important role, the SWI/SNF complex is conserved across species from yeast to humans, with homologous subunits.

Notably, 25% of cancers have mutations in at least one SWI/SNF subunit [11]. In this review, we will describe how various mutations in the SWI/SNF complex facilitate tumorigenesis and also examine the vulnerabilities of these cancers that can be targeted for treatment. However, to fully understand the implications of SWI/SNF mutations in cancer, a prologue is essential to understand what proteins constitute the SWI/SNF complex, what are their functions, and how the complex is assembled.

## Composition of the SWI/SNF chromatin remodeling complex

**Fig. 1:**
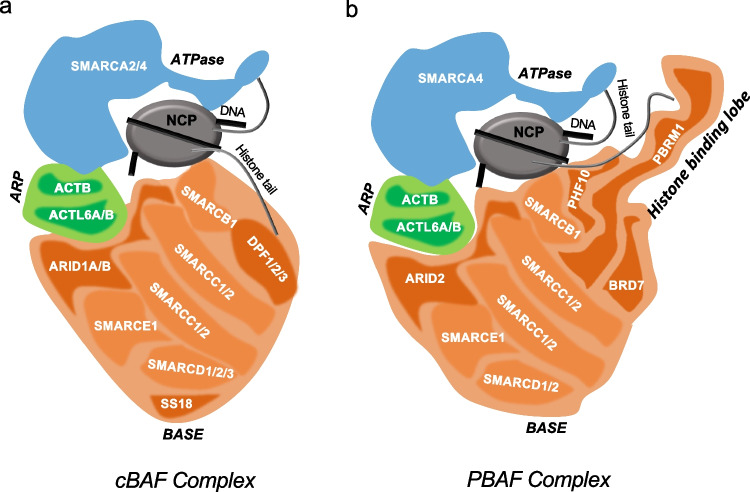
Mode of binding of SWI/SNF complex to nucleosome. Cartoon illustrating the binding of canonical BAF (cBAF) (**a**) and polybromo-associated BAF (PBAF) (**b**) with nucleosome core particle (NCP). The distinct modules are marked in distinct colors: blue for ATPase, green for ARP, and orange for base modules. Also, the unique subunits within the base modules are highlighted in darker orange color. PBAF complex uniquely contains a histone binding lobe which enables its interaction with modified histone tails in the NCP

The mammalian SWI/SNF (mSWI/SNF) complex is also called the BAF complex. It consists of a total of 12–15 subunits encoded by 29 genes, including multiple paralogs [12]. BAF complexes typically contain one of two mutually exclusive catalytic subunits, SMARCA4 or SMARCA2, as well as several additional/accessory subunits [12] (Fig. 1). The ATPase subunits not only contain the catalytic domain but also a bromodomain, and an AT-hook which enables their interaction with the substrate–nucleosome core particle (NCP) [13], while the accessory subunits either play a role in maintaining the integrity and function of the complex and/or have certain essential domains, crucial for the complex targeting to chromatin.

SMARCC1/2 and SMARCD1/2/3 associate to form a structural matrix, on which various other proteins are assembled [14]. Subunits SMARCB1, SMARCE1, and ARID family proteins—ARID1A/1B and ARID2—contain regions that mediate their interaction with transcription factors or DNA [15–17]. For example, the HMG (high mobility group) and ARID (AT-rich interaction DNA) domains in SMARCE1 and ARID proteins, respectively, enable the complex interaction with DNA [16, 17]. Similarly, the N and C terminus of SMARCB1 enable its interaction with transcription factors and NCP, respectively [15]. The transactivation domain in SS18/L aids its interaction with transcription activators [18]. A few other subunits—BRD7, 9, PBRM1 (Polybromo 1), and DPF1/2/3 and PHF10—have bromodomains and PHD (plant homeodomain), respectively, which enables the recognition of acetylated/methylated histone tails [19]. Actin B (ACTB) and ACTL6A subunits form an ATP-binding cleft required for maximal ATPase activity of SMARCA4 [20], while few other subunits like B-cell CLL/lymphoma 7 (BCL7) protein family members a/b/c, BCL11, and Glioma tumor suppressor candidate region gene 1/L (GLTSCR1/L) do not have any unique structured or functional domains and their role in SWI/SNF is largely unknown.

**Fig. 2:**
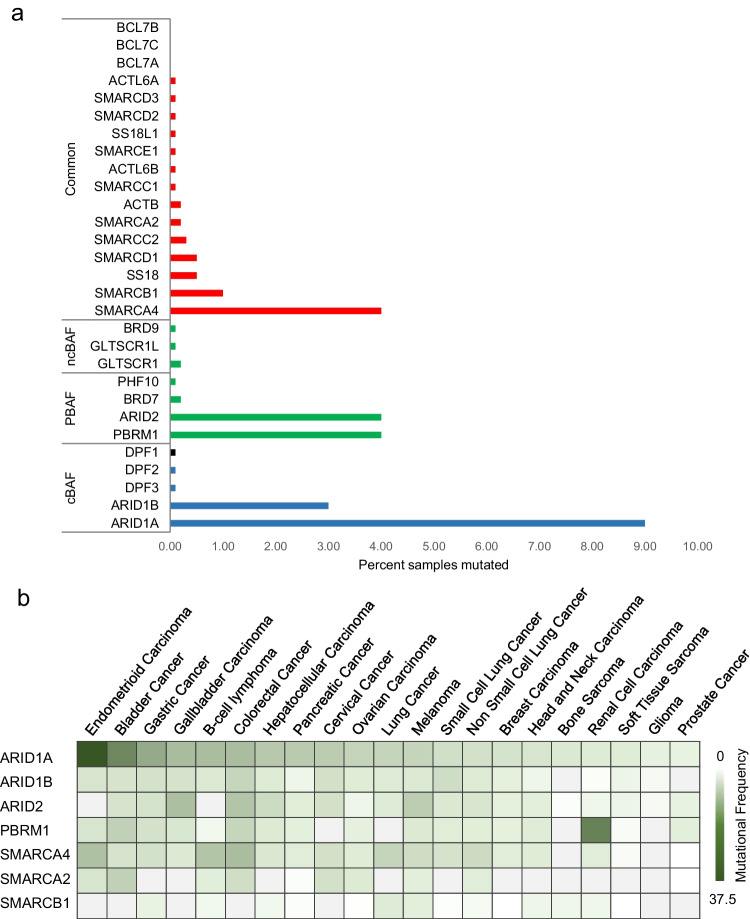
Mutational frequencies of SWI/SNF components in cancer. **a** Percentage of mutated samples for each SWI/SNF subunit was plotted by mining the data from two cancer datasets—Pan-Cancer Analysis of Whole Genomes and China pan-cancer dataset (*N* = 13,116) from The Cancer Genome Atlas (TCGA). The graph distinguishes various complex specific and shared subunits between cBAF, PBAF, and ncBAF complexes. **b** Heatmap of the frequency of a few frequently mutated subunits across various cancer types. The TCGA data set (*N* = 13,116) was used to generate the heatmap. The map also depicts that ARID1A is frequently mutated in various cancers, while PBRM1 is most mutated in renal cell carcinoma

Due to the presence of multiple subunits and paralogs, there could be hypothetically around 1500 different types of SWI/SNF complexes in a cell. However, there are three major biochemically distinct SWI/SNF complex assemblies—canonical BAF (cBAF), polybromo BAF (PBAF), and non-canonical BAF (ncBAF) [21, 22]. All three complexes share a few common subunits including the ATPase SMARCA4/2, but they also harbor a few exclusive protein components (Figs. 1, 2a). cBAF is the most abundant complex, around 1 MDa in size, and harbors two unique subunits—ARID1A/1B and DPF1/2/3 [21]. At the same time, PBAF is less abundant and contains ARID2, PBRM1, PHF10, and BRD7 as distinct subunits, whereas ncBAF (also called GBAF) is the smallest complex around 870 kDa in size, has GLTSCR1/L and BRD9 as two distinct protein components, and also does not contain cBAF subunits like SMARCB1, ARID, and SMARCE1 [21]. Interestingly, ncBAF complex subunit GLTSCR1 is present only in multicellular eukaryotes [23].

## Biochemical variations enable functional complexity in higher eukaryotes

Heterogeneity in SWI/SNF subunit composition enables heterogeneity in its function allowing SWI/SNF complexes to contribute to transcriptional regulation across cell types and stages of development. For example, SWI/SNF complexes contribute to the development of T cells, hepatocytes, oligodendrocytes, and the maintenance of embryonic stem cell self-renewal and pluripotency [24–27].

Specificity in the control of these developmental programs is achieved in part through restricted expression and combinatorial assembly of various SWI/SNF subunits and their paralogs. For instance, the SMARCD3 subunit is expressed specifically in the embryonic heart, where it regulates the heart-specific enhancers essential for the control of cardiac development [28]. Similarly, a switch from the PHF10 and ACTL6A subunits, expressed in neural stem cells, to DPF1, DPF3, and ACTL6B subunits are essential for the transition of proliferating neural progenitors into differentiation to post-mitotic mature neurons [29]. Subunit switching can modulate interaction with specific transcription factors and facilitates differential activation of transcriptional pathways. Furthermore, the careful balance between gene activation and repression is also achieved in part through BAF complexes opposing polycomb-mediated repression [30–33]. BAF complex recruitment leads to the rapid eviction of polycomb repressive complexes (PRCs) and their associated histone modification marks in the absence of Pol II occupancy, transcription, and replication [34]. The recruitment of SWI/SNF complexes to target genes largely happens (1) by the direct protein–protein interactions between various subunits and transcription activators [35–37], (2) by the direct interaction of SWI/SNF with modified histones due to the numerous interaction domains of the SWI/SNF subunits [38], and (3) indirectly by interacting with other transcriptional apparatus like histone acetyltransferases [36]. The recruitment then ultimately governs gene expression in various cellular pathways.

## SWI/SNF complex has distinct structural and functional modules

BAF complexes contain many subunits, which function together to bring changes in gene expression, but how these subunits work together to achieve this common goal has been a question of great interest. Recent biochemical and structural studies highlight that the mSWI/SNF subunits are assembled into distinct modules essential for the functioning of the complex [7, 21, 39, 40]. The complex is arranged in a “C shape clamp” around the nucleosome core particle (NCP) (Fig. 1). Their organization and interaction with the NCP suggest that there are three functional modules in the cBAF complexes. The *ATPase module* consists of the SMARCA4 subunit, which interacts with the topmost part of the NCP. The bulk of the *base module* is made up of SMARCB1, SMARCC, SMARCD, SMARCE1, and DPF subunits, where SMARCB1 interacts with the acidic patch of the NCP at the bottom. The subunits ACTB and ACTL6A form the regulatory *ARP (actin-related protein) module* connecting the ATPase and the base modules. This connection is further strengthened by the association of ARID1, which also stabilizes the base module binding to the NCP (Fig. 1a). The bilateral nucleosome engagement by SMARCB1 and SMARCA4/2 is essential as any deletion in the sequences interacting with NCP disrupts the maximum chromatin-remodeling activity of the cBAF complex [40, 41]. Interestingly, the mode of interaction and function of the PBAF complex with NCP is also similar with few changes; the base module in PBAF additionally has PBRM1, PHF10, BRD7, and ARID2 proteins. The role of ARID2 is highly similar to ARID1, although the rest of the subunits form a histone-binding lobe unique to PBAF and this probably enables its genome recruitment via sensing the chromatin environment (Fig. 1b). The three distinct complexes also show a varying degree of remodeling activity toward various modified nucleosomes [38]. Poly-acetylation of H3 tails activates all three complexes, while H4 acetylation selectively promotes the binding and activity of ncBAF, while H3K4me3 selectively inhibits cBAF activity while having minimal impact on PBAF and ncBAF remodeling activities [38]. The differential remodeling activities also may be the reason behind the distinct genome enrichment of the three complexes at specific chromatin signatures. cBAF complexes occupy mostly the active enhancer sites marked by the presence of H3K27ac and H3K4me1 suggesting their role in enhancer regulation [42, 43]. In contrast, PBAF is predominantly localized to active promoters harboring H3K27ac and H3K4me3 marks. Finally, ncBAF complexes are mostly enriched at CTCF sites co-localized with H3K4me1 [43].

## Cancer and SWI/SNF complexes

Advances in cancer genome sequencing revealed that SWI/SNF complex subunits are highly mutated in various cancers with a cumulative frequency approaching 25%, even higher than that seen for the tumor suppressor p53 [44]. In addition to the mutations within the complex subunits, their expression changes, and any mutations in the proteins enabling their interaction with chromatin can also have a direct or indirect regulatory effect on the functioning of the complex.

Large-scale pan-cancer genome-sequencing studies have reported that mutations occur across most of the genes encoding SWI/SNF subunits (Fig. 2a) [45, 46]. These mutations are widespread across various cancer types (Fig. 2b). They include nonsense, missense, frameshift, and deletion, which occur across the entire length of the genes. However, the most common type of mutation is missense mutations which are frequently located in the conserved domains of the SWI/SNF subunits [47, 48]. These mutations usually can lead to the degradation of the complex and, thus, loss from its target sites and/or formation of aberrant complexes that have either gain- or loss-of-function phenotypes. Interestingly, certain rare cancers like synovial sarcoma (SS), malignant rhabdoid tumor (MRT), and clear-cell meningioma are known to contain very few other genetic mutations apart from in SWI/SNF subunits [49–51]. This suggests that in these cancers SWI/SNF alterations have the potential to be driver mutations by giving a significant advantage for tumor initiation or growth [11]. Of the many subunits of the SWI/SNF complex, mainly five—SMARCA4/2, SMARCB1, ARID1A/B, PBRM1, and ARID2—are significantly mutated compared to the others (Fig. 2a) [52].

## Mutations in SWI/SNF subunits

### SMARCA4/2

SMARCA4 the ATPase is a common subunit with all three BAF complexes, and it is also frequently mutated in various cancers including breast, lung, and colorectal cancers [53–55]. It has been identified as a major tumor suppressor in various pan-cancer studies [56]. Interestingly, many of these mutations are missense and heterozygous in nature [48]. Furthermore, half of them occur within the conserved domains and, hence, potentially affect the function of the complex [48]. Therefore, these mutations can be subcategorized as loss- and gain-of-function mutations [57]. The former impairs DNA translocation, thereby hindering chromatin remodeling (inactivates) whereas the latter increases DNA translocation efficiency, nucleosome remodeling, and hence, chromatin accessibility (hyperactivates) [57].

Accordingly, heterozygous expression of SMARCA4 also mimics a dominant negative phenotype, as DNA accessibility at various enhancers is lost inducing pro-oncogenic expression changes via the MYC signaling pathway [48]. Another way these mutations bring about their effect is by increasing the chromatin retention of Polycomb repressive complex (PRC) complexes, leading to H3K27me3 changes at CpG-island promoters and contributing indirectly to tumor development [58]. SMARCA4 mutations are also found in genetically complex tumors, presumably adding to their genetic burden. These mutations can act as progression events and usually correlate with a poorer prognosis as observed with non-small cell lung cancer [54]. Elevated SMARCA4 expression has also been reported in many cancers including colorectal, gastric, prostate, and intestinal cancers [59–63].

Unlike SMARCA4, its paralog SMARCA2 is not frequently mutated in cancers but rather is epigenetically silenced across numerous tumor types and cancer cell lines [64]. The exact mechanism of epigenetic silencing varies but can arise through promoter methylation, polymorphisms, or HDAC/EZH2-driven mechanisms [65–68]. Furthermore, its transcriptional reactivation by knockdown or use of HDAC inhibitors prevents cell proliferation [69]. In a certain subset of tumors like small cell carcinoma of the ovary, hypercalcemic type (SCCOHT), both ATPases are not expressed [70, 71]. Loss of both the catalytic subunits leads to a formation of a residual SWI/SNF complex which still can bind to chromatin but with less affinity; however, there is no formation of distinct cBAF and PBAF complexes [72]. The reintroduction of ATPase-deficient SMARCA4 in SCCOHT cell lines restores complex localization to a few enhancers and promoters, but does not promote tumor-suppressive gene expression programs, suggesting that the catalytic activity is essential for mediating these functions [72]. In SMARCA4-deficient cancers that retain SMARCA2 expression, several reports have shown that SMARCA2 acts as a synthetic lethal target, making it a potential therapeutic vulnerability [48, 49].

### SMARCB1

SMARCB1 is present in both cBAF and PBAF complexes. The discovery of mutations in the SMARCB1 gene in rhabdoid tumors in 1998 was the first evidence linking the SWI/SNF complex to cancer [73]. Beyond the mutations in SMARCB1 which lead to the loss of its expression, these tumors are genetically simple, bearing no other frequent driver mutations [74]. Studies in mouse models demonstrate that homozygous inactivation of SMARCB1 is embryonically lethal, although induced somatic homozygous loss results in the rapid onset of cancer in 100% of mice at 11 weeks [75–77]. This remarkably takes half the time required for tumor formation following P53 inactivation [78].

Heterozygous mutations cause rhabdoid tumor formation in 10–30% of mice, suggesting that SMARCB1 is a bonafide tumor suppressor [75–77]. Mechanistically, the loss of SMARCB1 protein affects the integrity of the SWI/SNF complex and also dissociates the SWI/SNF complex from chromatin [79, 80]. This probably is due to the direct interaction of SMARCB1 with the NCP, necessary for the proper functioning of the BAF and PBAF complexes as discussed above. However, it is the residual BAF complexes deficient in SMARCB1 which drive tumor formation. These aberrant complexes are unable to evict Polycomb, resulting in an elevated H3K27me3 mark, especially at the p16Ink4a tumor suppressor locus thus driving oncogenesis in malignant rhabdoid tumors [81, 82].

Not only mutations but expression changes have also been reported for SMARCB1 in cancer. It is highly upregulated in hepatocellular carcinoma (HCC), leading to the expression of Nucleoporin 210 (NUP210), important for xenobiotic metabolism. This, in turn, promotes tumor formation and is therefore associated with a poor prognosis of the disease [83]. A reduced expression of SMARCB1 has been reported for synovial sarcoma (SS). The translocation of 78 amino acids of SSX at a locus of SS18, an SWI/SNF subunit, leads to the expression of SS18–SSX fusion mutant protein [84, 85]. The fusion protein has been shown to integrate into the BAF complex and acts in a dominant negative manner, displacing not only the wild-type SS18 but also SMARCB1 [86, 87]. The dislocated SMARCB1 is immediately degraded, mimicking reduced SMARCB1 expression, a molecular signature associated with SS. This change in biochemical composition causes cBAF complex degradation and an increase in the prevalence of PBAF and ncBAF complexes [88]. These changes ultimately redirect the BAF complexes from enhancers to broad polycomb domains, activating bivalent genes and leading to oncogenesis [87].

### ARID1A/1B

ARID1A is a cBAF-specific subunit and the most frequently mutated SWI/SNF subunit across cancer types [11]. It was first noted for its mutation status in ovarian clear cell carcinoma, where it is mutated in nearly 60% of the cases [89, 90]. Most of the mutations are loss of function, with nonsense or frameshift mutations occurring all through the gene length. Furthermore, the knockdown of ARID1A is enough for the malignant transformation of immortalized endometrial cells, suggestive of its role as a tumor suppressor [91]. However, studies using mice models suggest that the role of ARID1A in cancer is complex, and often context dependent, and it has both tumor-suppressive and oncogenic roles [42, 92]. Homozygous or heterozygous loss of ARID1A is tumor suppressive due to the decreased chromatin accessibility at enhancers and also decreased expression of genes linked to migration, invasion, and metastasis [42, 93]. Interestingly, elevated ARID1A levels promote tumor initiation by increasing oxidative stress through Cytochrome P450 pathways [93]. Although not that abundant, mutations in ARID1B are also identified in neuroblastoma and pancreatic cancer [94, 95]. Interestingly, mutations in the nuclear localization signal of ARID1B are observed in pancreatic cancer [96]. This leads to the cytoplasm localization of ARID1B which has been shown to promote oncogenesis by stimulating the ERK signaling pathway [96].

Dual mutations in the ARID1 paralogs have been reported in gastric, endometrial, and liver cancers [97]. Furthermore, more than 30% of the reported ARID1A mutant cell lines also harbor loss of ARID1B protein [98]. Mechanistically, dual loss leads to the formation of residual complexes, which associate with PBAF and disrupt its oligomerization [97]. This in turn affects PBAF chromatin distribution contributing to aggressive carcinogenesis in skin and liver mice models [42, 97].

### PBRM1 and ARID2

PBRM1 is a PBAF-specific subunit that contains six bromodomains (BD) [99]. It is mutated in 40% of clear-cell renal carcinoma tumors (ccRCCs) [100]. After VHL, PBRM1 is the second most frequently mutated gene in ccRCC. The combined loss of VHL and PBRM1 is necessary and sufficient for renal malignancy as demonstrated by genetic mouse models [101]. Together, they govern hypoxia gene expression and relieve cells of replication stress [100, 102]. The mutations in PBRM1 often cause loss of protein expression, although few missense mutations are concentrated in the bromodomains [99]. Mutations in the BD2 and BD4 disrupt the chromatin binding of PBRM1 *in vitro*, negatively affecting gene expression pathways necessary for cell proliferation *in vivo* [99, 103]. These mutations probably influence the histone-binding lobe of the PBAF complex, rendering it incapable to bind to acetylated chromatin.

ARID2 is also a PBAF-specific subunit and acts similarly to ARID1. It is frequently mutated in HCC, melanoma, ovarian, breast, and lung cancers [104–106]. In liver cells, ARID2 enables the transcription of interferon (IFN)-γ [107]. Therefore, the loss of ARID2 leads to disruption in IFN-γ signaling pathways essential for the maintenance of a tumor-suppressive environment in hepatocytes [107].

Studies across various SWI/SNF subunit mutations in many cancers reveal a common theme where these mutations inactivate or destabilize the complex. This ultimately affects the gene regulatory pathways in place to prevent oncogenesis. However, the superimposition of various common mutations on the structure of the BAF complex suggests that only 44% account for their role in complex formation and stability [108]. This suggests that there could be additional mechanisms concerning how these mutations affect the complex function. These mechanisms might include various mutations on the subunits, influencing their binding to a transcription factor, or changes in post-translational modifications manipulating the binding activity of the complex. For example, mutations in BD4 of PBRM1 promote tumor progression by disrupting P53 transcriptional activity and by failing to recruit acetylated P53 at its target promoters [109]. Furthermore, not only mutations within the SWI/SNF complex subunits but any mutations/alteration in its interacting partners essential for the complex recruitment to chromatin also can lead to disruption in its genome distribution promoting tumor conducive environment.

## Mutations in the interaction partners of SWI/SNF subunits

Studies done in yeast have identified that the binding of SWI/SNF to chromatin is largely dependent on its association with various transcription factors (TFs) [110, 111]. Genetic alterations in these TFs may also play an important role in many tumor types in which SWI/SNF subunits are not genetically altered, thereby expanding the already wide-spanning role of these complexes in human cancer.

Many tumor suppressors proteins like TP53, MYC, Retinoblastoma (Rb), and BRCA1 interact with various SWI/SNF subunits [112–118]. Furthermore, more than 40% of P53 mutants bring about their effect by modulating SWI/SNF activity or recruitment to chromatin targets [117, 119, 120]. Similarly, MYC binds to various components of SWI/SNF and also regulates the expression of SWI/SNF subunits themselves [112, 113]. In addition, as SMARCB1 has a binding preference to the acidic patch of the nucleosome, any mutations in the histones also affect the binding and remodeling activities of SWI/SNF [121–123].

SWI/SNF complexes also display a gain-of-function activity in cancers harboring chromosomal translocations leading to the expression of oncofusion TFs. Particularly, FET (EWSR1::FLI1, FUS::DDIT3) and ETS (TMPRSS2::ERG) oncofusions’ role in perturbing SWI/SNF complex genome binding has been noted. EWSR1::FLI1, found in Ewing sarcoma (EWS), gains the interaction with the SWI/SNF complex and guides it to genes having GGAA microsatellite repeats, enabling oncogenic gene transcription [124, 125]. Similarly, TMPRSS2::ERG, occurring in approximately 50% of prostate cancer cases, gains interaction with the SWI/SNF complex and retargets it from AR to ETS sites [126–128]. This ultimately facilitates basal to the luminal transition of cells which is essential for prostate cancer progression [128, 129]. Unlike these two fusion proteins that direct the targeting of SWI/SNF complexes to loci that support oncogenic gene expression and proliferation, FUS::DDIT3 binding uniquely acts as a loss-of-function mutation. FUS::DDIT3 is found in 95% of myxoid liposarcoma (MLS), binds to SWI/SNF and prevents its binding at adipogenic enhancers, and upregulates tumorigenic pathways [130–132].

Collectively, the mutations and aberrant expression in SWI/SNF subunits or their interactors may contribute to disease progression in even more than 25% of the cancers than initially anticipated.

## Therapeutic modalities for targeting SWI/SNF-altered cancers

Systemic investigation on SWI/SNF mutant cancer lines using shRNA and CRISPR libraries has identified several promising candidates for targeted therapy of SWI/SNF-altered cancers. These genes not only include other SWI/SNF subunits but also their interactors. Furthermore, several of these vulnerabilities are being pursued in their therapeutic translation, and a few of these approaches are being tested in ongoing clinical trials.

### Targeting intracomplex vulnerabilities

One vulnerability that emerges in cancers is the mutations in a few SWI/SNF subunits that lead to specific dependency on other SWI/SNF genes. For example, the loss of ARID1A in ovarian and colorectal cancers creates a dependency on its paralog, ARID1B [98, 133]. Similarly, SMARCA4 mutant cells show an enriched dependency on SMARCA2 [134, 135]. This is due to the compensatory mechanism inside the cells. Nevertheless, there are cancers having mutations in both the paralogous subunits of SWI/SNF, but these are rare [71]. Therefore, the development of specific chemical degraders such as PROTACs makes this approach tractable [136–138]. *Pro*teolysis *ta*rgeting *c*himeras (PROTACs) use structure-based design to direct E3 ubiquitin ligases to the specific protein of interest for their degradation [136]. ACBI1 is a bifunctional degrader developed specifically against the bromodomain of SMARCA4 and SMARCA2, inducing their degradation [139]. Treatment with this degrader led to cell death in SMARCA4 mutant cell lines [139]. PROTACs are highly specific, and accordingly, ACBI1 selectively degrades only SMARCA2 and SMARCA4 and does not show any effect on other bromodomain-containing proteins [139]. Furthermore, recently developed ACBI2 preferentially degrades SMARCA2 and induces lung cancer tumor growth inhibition [140].

Small molecule inhibitor PFI-3 has been developed against the bromodomains of SMARCA2, SMARCA4, and PBRM1 [141, 142]. However, it was an ineffective treatment for SMARCA4 mutant cell lines [141]. Furthermore, cDNA complementation studies suggest that the inhibition of SMARCA2 ATPase activity has a negative influence on the growth of SMARCA4 mutant cells [141]. Accordingly, dual ATPase inhibitors for SMARCA2 and SMARCA4 have been developed and tested for their antitumor activity in cells deficient in SMARCA4 [143]. In addition, such dependencies are also reported between SMARCC1/SMARCC2, SMARCA4/ACTB, and SMARCA4/ARID2, although they are yet to be functionally tested before developing targeted strategies [144].

### Targeting inter-complex vulnerabilities

Screening for genetic and/or pharmacological vulnerabilities in various SWI/SNF-altered cancer cell lines has not only yielded new mechanistic insights into the functioning of the complex but also revealed a new group of targets, which should be further explored for their therapeutic potential.

PRC2 and mSWI/SNF complexes have opposing effects on gene expression [81]. Furthermore, mutations in SMARCB1 and SMARCA4 lead to widespread changes in the H3K27Me3 distribution, changing gene expression patterns, and suggesting that PRC2 can be targeted in these cancers [58, 81, 82]. Furthermore, SWI/SNF mutant cell lines and xenograft models are particularly dependent on PRC2 activity, and its inhibition suppresses the oncogenic signaling [145]. The use of EZH2 (PRC2 catalytic subunit)-specific chemical inhibitor tazemetostat has been approved for the treatment of MRT and epithelioid sarcomas [146, 147]. The efficacy of this treatment is being currently tested in other cancers having SMARCB1 or SMARCA4 mutations [148, 149]. In addition, alternative strategies are also being tested to inhibit PRC2 with newly developed inhibitors targeting other components of the PRC2 complex [149, 150]. Inhibition of EZH2 has also been shown to be lethal for ARID1A mutant ovarian cancer cells due to the activation of the PI3K/AKT signaling pathway [151]. On the other hand, the ATPase switch from SMARCA4 to SMARCA2 during the EZH2 treatment can lead to acquired resistance, which also accompanies BCL2 upregulation [152]. BCL2 is an anti-apoptotic gene, which can be inactivated by the use of its inhibitor ABT263 [153]. Therefore, a combination treatment of inhibiting BCL2 and EZH2 is a better therapeutic strategy for ARID1A mutated cancers.

Treatment with pan-HDAC inhibitor panobinostat or SAHA induces cellular senescence in SMARCB1 mutant rhabdoid and ovarian tumor cells [154, 155], while ARID1A-deficient tumors rely on HDAC6 activity for the deacetylation of P53, required for repression of pro-apoptotic genes [156]. The use of ricolinostat, an HDAC6-specific inhibitor, increases P53 acetylation, promotes apoptotic response, and improves the survival of mice harboring ARID1A mutant cancer [156].

SMARCA4 inactivating mutations in lung cancers increase their sensitivity to CDK4/6 and Aurora Kinase inhibition [157, 158]. Interestingly, ARID1A mutations co-occur with PIK3CA mutations [159]. Loss of ARID1A in breast cancer cells activates AKT; furthermore, treating these cells with MK-2206 (AKT inhibitor) and buparlisib (PI3K inhibitor) increased apoptosis [160]. A similar effect was also observed in various ARID1A mutant ovarian cancer cell lines and mice [159]. Therefore, ARID1A mutant cancers are highly susceptible to the inhibition of PI3K and AKT kinases.

The role of ARID1A in regulating genome stability by recruiting DNA repair proteins like MSH2 at damage sites is known [161, 162]. Accordingly, the loss of ARID1A makes cells sensitive to DNA-damaging agents like radiation, ATR, and PARP inhibitors [163, 164]. Xenograft mice having ARID1A tumors show a drastic decrease in tumor burden upon combined radiation and PARP inhibitor treatment [164]. Therefore, several clinical trials are underway in patients with ARID1A mutant cancers with inhibitors of ATR and PARP (ClinicalTrials.gov Identifiers NCT03207347, NCT04042831, NCT02576444, NCT04065269). Similarly, SMARCA4- and PBRM1-deficient tumors also show a sensitivity to ATR and PARP inhibition [165–167].

Large-scale CRISPR screening, aimed at understanding the genetic elements enabling T-cell-mediated tumor killing of melanoma cells, revealed the role of the PBAF complex in immune cell signaling pathways [168]. Depletion of PBRM1, BRD7, ARID2, or any of the PBAF-specific components enhances T-cell response against cancer cells in mouse xenograft models [168]. Accordingly, treatment of ccRCC patients with anti-programmed cell death 1 (PD-1), an immune checkpoint inhibitor, positively correlates with reduced tumor burden and therefore is clinically beneficial [169, 170]. A similar observation has also been made in mice bearing ARID1A-deficient ovarian and gastric cancer [162, 171].

Metabolic vulnerabilities have also been reported for BAF mutant cancers. Especially, ARID1A is shown essential for maintaining glutathione homeostasis by promoting the expression of cystine transporter, SLC7A11 [172]. Hence, ARID1A mutant cells are particularly sensitive to inhibition of the GSH metabolic pathway [173, 174]. Treatment with APR-246 (which targets GSH) and buthionine sulfoximine (for glutamate–cysteine ligase synthetase catalytic subunit) leads to apoptotic cell death due to elevated reactive oxygen species [172]. SMARCB1-deficient tumors show sensitivity to inhibition of the proteasome machinery and autophagy pathways [175], while SMARCA4 mutated lung cancer cells have increased oxygen consumption and enhanced respiratory capacity. Therefore, they are sensitive to IACS-010759, an oxidative phosphorylation (OXPHOS) inhibitor [176].

These studies demonstrate clearly that there are multiple prospects available for targeting SWI/SNF mutant tumors. However, a detailed investigation in both pre-clinical models, as well as large-scale patient trials, is essential for understanding which options are tractable, and this should be the focus in the future.

## Conclusion

In yeast, SW/SNF complex regulates the expression of around 5% of the genome. In humans, nonetheless, they seem to have a much more enormous impact on chromatin structure and therefore regulate almost all the cellular pathways. Recent discoveries on how the complex assembles, its 3D structure, TF interactions, and genome-wide distribution in wild-type and mutant cells have revealed intricate mechanistic details of the working of the complex. This has also revealed the basis of oncogenesis in SWI/SNF mutated cancers.

SWI/SNF subunits are mutated at a collective frequency of 25% in all cancers. However, considering the broad role of the complex and its interactors in various physiological pathways, by both direct and indirect mechanisms, SWI/SNF may be affected at much higher frequency in cancers. Many mutations in various subunits display cell line– or tissue-specific dependency; therefore, there is a need for the development of tailor-made therapies for individual cancer types. However, elucidating the distinct functions and networks of SWI/SNF complex members across various cancer types as well as developmental stages will enable the development of more holistic therapies targeting a wide variety of cancers.
